# Utility-based optimization of Fujikawa’s basket trial design – Pre-specified protocol of a comparison study

**DOI:** 10.1371/journal.pone.0323097

**Published:** 2025-05-28

**Authors:** Lukas D. Sauer, Alexander Ritz, Meinhard Kieser

**Affiliations:** 1 Institute of Medical Biometry, Heidelberg University, Heidelberg, Germany; 2 Institute of Mathematics, Clausthal University of Technology, Clausthal-Zellerfeld, Germany; University of Connecticut School of Medicine, UNITED STATES OF AMERICA

## Abstract

Basket trial designs are a type of master protocol in which the same therapy is tested in several strata of the patient cohort. Many basket trial designs implement borrowing mechanisms. These allow sharing information between similar strata with the goal of increasing power in responsive strata while at the same time constraining type-I error inflation to a bearable threshold. These borrowing mechanisms can be tuned using numerical tuning parameters. The optimal choice of these tuning parameters is subject to research. In a comparison study using simulations and numerical calculations, we are planning to investigate the use of utility functions for quantifying the compromise between power and type-I error inflation and the use of numerical optimization algorithms for optimizing these functions. The present document is the protocol of this comparison study, defining each step of the study in accordance with the ADEMP scheme for pre-specification of simulation studies.

## 1 Introduction

With the dawn of precision medicine and targeted antibody therapies, the wish for more flexible trial designs compared to randomized controlled trials has been emphasized in both clinical research and methodology. While randomized controlled trials are still the gold standard of clinical research owing to their high internal validity, in some contexts they may be costly, slow, unethical, or simply not feasible. The term *master protocols* summarizes more flexible trial designs that combine features such as the addition of promising new treatment arms, the removal of ineffective treatment arms, and the combination of arms with different endpoints. A commonly requested idea is testing the same treatment in several substrata of a patient cohort. Such a master protocol, i.e., a design that unifies several strata in a single clinical trial, is called *basket trial design*. This unification streamlines the planning phase of the different strata and parallelizes their recruitment, resulting in an efficient use of resources. Basket trial designs are beginning to be picked up in practice. A systematic literature review conducted on February 20, 2023, in MEDLINE, Embase, and the Cochrane Central Register of Controlled Trials found 146 oncology-related basket trials [1]. Especially in early stages of clinical research, these designs also offer statistical benefits: So-called *borrowing* techniques allow strata with similar responses to the treatment to share information with one another, thereby leveraging power despite small sample sizes while keeping type-I error rates only moderately inflated. The earliest publication known to us that suggests borrowing between strata was published in 2003 [2] and the last ten years showed a colorful bouquet of Bayesian and frequentist borrowing mechanisms being introduced to methodological research [3].

Borrowing usually depends on the choice of several tuning parameters. An optimal choice of tuning parameters has to offer a compromise between multiple components: achieving high power in responsive strata, keeping type-I error rates low in unresponsive strata and maintaining a good balance between these two measures across several response scenarios. This compromise can be quantified using utility functions. The optimal choice of tuning parameters can then be found by finding optimal utility function values with the help of numerical optimization algorithms. In the following document, we present the protocol of a comparison study planned to investigate utility-based optimization of basket trial designs using both simulations and numerical calculations. The basket trial design that we are considering as an example is a Bayesian design introduced by [4].

## 2 Methodology of utility functions in basket trial designs

In the statistical planning of clinical trials, the communication of type-I error rate (TOER) and power to stakeholders such as principal investigators, sponsors, ethical committees and regulatory authorities is essential. TOER is the probability of rejecting the null hypothesis conditional on the assumption that the null hypothesis is true. Power is the probability of rejecting the null hypothesis conditional on the assumption that some alternative hypothesis of interest holds. While these measures are purely frequentist in nature, they may also be requested during the planning of Bayesian trial designs.

In the planning of basket trial designs, this demand for TOER and power calculation is confronted with several challenges. Firstly, every stratum may have its own null and alternative hypothesis of interest so that a multitude of combinations of null and alternative hypotheses across baskets can be considered. Secondly, control of TOER in a scenario may not be possible if we want to employ borrowing in order to leverage power. [5] proved that power increase always comes at the cost of TOER inflation in the context of borrowing from external data sources whenever a uniformly most powerful test exists. While we are not aware of a formal transfer of their argument to the context of basket trials, it is plausible that the argument holds in that setting as well.

In communication with stakeholders, the best practice may be to communicate both TOER and power for each stratum across a range of plausible scenarios combining null and alternative hypotheses in different strata.

However, when searching for the optimal choice of tuning parameters of a basket trial designs we need to combine TOER and power across strata and scenarios. A natural way of combining these values is by defining an appropriate utility function. Then, optimization algorithms can be employed in order to find the optimal tuning parameter vector. Constraints, e.g. the maximally tolerated TOER values, may either be incorporated into the utility function allowing for unconstrained optimization methods or may be set up as separate inequalities, asking for constrained optimization approaches. This utility-based optimization approach has already been employed in the context of adaptive designs [6] and in the context of planning several stages of drug development [7] even though formal control of TOER is possible in these contexts. In the methodology of basket trial designs, we are aware of a first utility-based approach presented in [8]. Their approach will also be considered in the presented comparison study protocol and will be supplemented by several other optimization algorithms and utility functions.

## 3 Goals of this comparison study

This comparison study’s goal is to find optimal tuning parameter combinations for Fujikawa’s basket trial design in a general framework that could subsequently be generalized to other basket trial designs as well. In particular, the study is divided into three parts addressing three questions related to finding optimal parameter combinations:

1. Which type of optimization algorithm for finding the optimal tuning parameter vector $\phi^{*}$ should be preferred in terms of runtime and reliability?
2. What is a good definition of *optimal* tuning parameter vector $\phi^{*}$ that takes the desire for maximizing the detection probability of active strata as well constraints on TOER into account while delivering favorable results across a range of outcome scenarios? This question amounts to finding an appropriate utility function. We will use the best algorithm found in Part I, and apply it to a variety of different utility functions.
3. How does the optimal tuning parameter vector $\phi^{*}$ found as a result of parts I and II perform in comparison to the tuning parameter combinations suggested in [4]?

The document will begin with a brief introduction into basket trial designs in general and into Fujikawa’s design in particular. Afterwards, we will explain the true scenarios of interest in our study as well as the considered utility function utility functions and optimization algorithms. Then we will present the pre-specified plan of the three parts of the study structured using the ADEMP scheme by [9]. Part I is a simulation study for comparing different algorithms, parts II and III are comparison studies where all measures of interest can be calculated exactly.

## 4 Basket trial designs

A basket trial design is a clinical trial design used primarily in oncological single-arm phase II studies. It tests the same null hypothesis in several strata. In the literature, either the ensemble of all strata together is called “basket” or the strata themselves are called “baskets”. In the following, the endpoint will always be binary. While this could be any binary endpoint, we will without loss of generality refer to response to a treatment vs. no response throughout the text. Strata that have a sufficiently high true response rate to the treatment are called *active*, otherwise they are called *inactive*. In particular, the primary comparison is against a specified target rate and not against a control group. As no control group exists, the designs we consider are stratified single-arm trials and not multi-arm trials.

Consider the design from [4], which is based on an alteration of the beta-binomial model. Using the notation from [3], it can be defined as follows: Let *n*_*i*_ resp. *r*_*i*_ be the number of patients resp. responders in stratum i∈1,…,I for some number of strata *I*. The sampling distribution is simply the binomial distribution


$$r_{i}\sim Bin(n_{i},p_{i}),$$

where the true rate *p*_*i*_ follows a prior beta distribution with shape parameters $a_{i},b_{i}>0$,


$$p_{i}\sim Beta(a_{i},b_{i}).$$

For analysis of data, Fujikawa *et al*. recommend an uninformative choice of prior distribution. We choose $a_{i}=b_{i}=1$. By conjugacy, the usual posterior distribution would be

$$p_{i}\sim Beta(a_{i}+r_{i},b_{i}+n_{i}-r_{i})={Beta}_{i}^{\text{post}}.$$ (1)

Now this posterior is altered by introducing a borrowing mechanism,

$$p_{i}\sim Beta(\sum_{j}\omega_{ij}\cdot (a_{j}+r_{j}),\sum_{j}\omega_{ij}\cdot (b_{j}+n_{j}-r_{j}))={Beta}_{i}^{\text{bor}}.$$ (2)

Here $\omega_{ij}$ is a similarity measure defined by $\omega_{ij}=1({\tilde{\omega }}_{ij}^{\varepsilon }>\tau )\;\cdot \;{\tilde{\omega }}_{ij}^{\varepsilon }$, where we have τ∈[0,1], ε≥0, and ${\tilde{\omega }}_{ij}=1$ − $JSD({Beta}_{i}^{\text{post}},{Beta}_{j}^{\text{post}})$ with the Jensen-Shannon divergence JSD of the unaltered beta-binomial posterior distributions from Eq 1. Note that in the original publication [4], the bound for ε is set to ε≥1. However, there is no mathematical or design-related reason to not allow values between 0 and 1. JSD is a measure of divergence of probability distributions which implies that $\omega_{ij}$ becomes a measure of similarity of probability distributions, which is set to 0 if the similarity is less or equal to τ. The Jensen-Shannon divergence is defined as


$$JSD(P,Q)=\frac{1}{2}(KLD(P,M)+KLD(Q,M)),$$

where $M=\frac{1}{2}(P\;+\;Q)$ is the mixture distribution of *P* and *Q* [4]. Here, KLD is the Kullback-Leibler divergence defined as


$$KLD(P,Q)=\int_{𝒳}P(x)\log\left(\frac{P(x)}{Q(x)}\right)\mu (dx),$$

where (X,S,μ) is the probability space on which *P*, *Q* or *M* are defined [10] and P(x) denotes the Radon-Nikodym derivative with respect to μ, i.e. the probability density function in case μ is chosen to be the Lebesgue measure. Here, log(·) denotes the natural logarithm. We use this logarithm for better comparability with Fujikawa’s results where the natural logarithm is used as well. In [11], the logarithm with base 2 is used as it implies that the Jensen-Shannon divergence ranges from 0 to 1.

The test decision whether stratum *i* is *detected* as active is based on the posterior probability of lying above a desired target rate $p_{i}^{*}$, i.e.,

$$P(p_{i}>p_{i}^{*}|𝐫)\geq \lambda ,$$ (3)

where $𝐫=(r_{i}{)}_{i}$ is the vector of responses and where the posterior probability P(·|𝐫) is defined with respect to the borrowing posterior ${Beta}_{i}^{\text{bor}}$.

We denote by ϕ=(λ,ε,τ) the vector of tuning parameters. In the examples of [4], the shape parameter ε, the similarity cutoff τ and the detection threshold λ are chosen to be either ϕ=(λ,ε,τ)=(0.99,2,0) or (0.99,2,0.5), but it is unclear whether this is the optimum for choosing the tuning parameters.

Concerning the relationship between ε and τ, one should note two things. Firstly, they are redundant when it comes to defining the minimal similarity for which borrowing is still allowed. Indeed, for a given minimal similarity ${\tilde{\omega }}^{*}$ and a given τ, we can choose $\varepsilon_{{\tilde{\omega }}^{*}}(\tau )=\log_{{\tilde{\omega }}^{*}}(\tau )$ (and analogously τω~*(ε)=(ω~*)ε) such that the function $\omega_{ij}=1({\tilde{\omega }}_{ij}^{\varepsilon }>\tau )\;\cdot \;{\tilde{\omega }}_{ij}^{\varepsilon }$ $\max_{r_{k}\neq r_{l}}{\tilde{\omega }}_{kl}$ is greater than 0 if and only if ${\tilde{\omega }}_{kl}>{\tilde{\omega }}^{*}$.

Secondly, this implies that when choosing ${\tilde{\omega }}^{*}=\max_{r_{i}\neq r_{j}}{\tilde{\omega }}_{i,j}$ or greater, then borrowing is only allowed for two substrata that have identical response rates. We call this the *extreme borrowing boundary* $\varepsilon_{\text{extreme}}(\tau )$. Increasing ε or τ above this boundary does not change the behavior as $\omega_{ij}$ is either equal to 1 in case $r_{i}=r_{j}$ or equal to 0 in case $r_{i}\neq r_{j}$.

**Fig 1:**
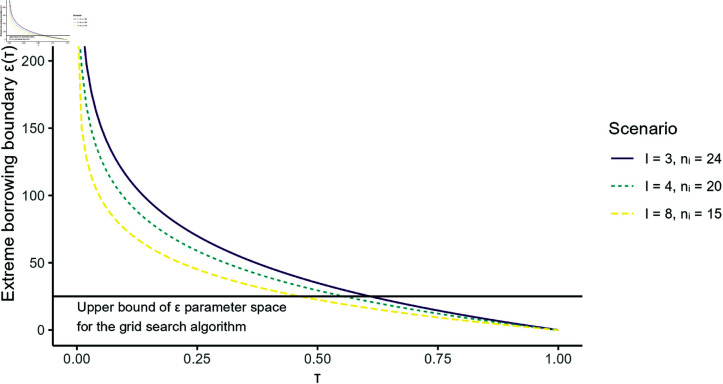
Extreme borrowing boundary of Fujikawa *et al*.’s tuning parameters. Extreme borrowing boundary $\varepsilon_{\text{extreme}}(\tau )$ for different stratum counts *I* and per-stratum patient counts *n*_*i*_ and the upper bound of the ε parameter space for the grid search algorithm.

The parameter space upon which we will perform optimization in this study will be λ∈[0,1], ε∈[0,∞) and τ∈[0,1]. For the grid search algorithm (explained below), we will restrict ourselves to ε∈[0,25]. This means that the parameter space will encompass the parameter suggestions by Fujikawa *et al*. and that we can study the behavior of the extreme borrowing boundary for higher τ values (approx. above τ=0.6) as can be seen in Fig 1.

## 5 Outcome scenarios

In order to study the performance of the tuning parameter combinations, each step of the comparison study will consider the outcome scenario sets summarized in Table 1. The outcome scenario sets differ in the number of strata (*I* = 3 to *I* = 20), in the response rates assumed for inactive and active strata (*p*_0_ = 0.01 to 0.20, *p*_1_ = 0.10 to 0.50) and in their total and per-stratum sample sizes (total sample sizes ranging from 72 to 480). With the aim of using scenarios that are representative of both relevant methodological research and actual clinical trials, we considered three methodological publications, [4, 12], and [11], four systematic reviews of basket trials, [13, 14, 15], and [1], and additional information from the website ClinicalTrials.gov for the study [16]. Details on the choice of scenario sets can be found in S1 File Section 1.

**Table 1:** Outcome scenarios sets.

*I*	$p_{0}$	$p_{1}$	$n_{i}$	Scenarios (from methodological paper/similar to registered trial)	Reference
3	0.20	0.50	24	Three-stratum scenarios from [4]eak $𝐩=(0.20,\ldots ,0.20,\underset{a}{\underbrace{0.50,\ldots ,0.50}})$ with 0≤a≤3	[4]
3	0.15	0.30	53	Large per-stratum sample size similar to NCT01631552eak $𝐩=(0.15,\ldots ,0.15,\underset{a}{\underbrace{0.30,\ldots ,0.30}})$ with 0≤a≤3	[1]
4	0.15	0.40	20	Four-stratum scenarios from [11]eak 𝐩=(0.4,0.4,0.3,0.5), “one in the middle”eak 𝐩=(0.15,0.25,0.35,0.45), “linear”eak $𝐩=(0.15,\ldots ,0.15,\underset{a}{\underbrace{0.40,\ldots ,0.40}})$ with 0≤a≤4eak In [11], these last five scenarios with 0 to 4 active strata are called global null, good nugget, half, bad nugget and global alternative.	[11]
4	0.10	0.35	36	Medium per-stratum sample size similar to NCT01848834eak $𝐩=(0.10,\ldots ,0.10,\underset{a}{\underbrace{0.35,\ldots ,0.35}})$ with 0≤a≤4	[16]
8	0.15	0.45	15	Eight-stratum scenarios analogous to [12]eak $𝐩=(0.15,\ldots ,0.15,\underset{a}{\underbrace{0.45,\ldots ,0.45}})$ with 0≤a≤8	[12]
9	0.01	0.10	23	Medium total sample size and small effect sizes, similar to NCT02454972eak $𝐩=(0.01,\ldots ,0.01,\underset{a}{\underbrace{0.10,\ldots ,0.10}})$ with 0≤a≤9	[1]
20	0.10	0.35	24	Large total sample size and large number of baskets, similar to NCT02054806eak $𝐩=(0.10,\ldots ,0.10,\underset{a}{\underbrace{0.35,\ldots ,0.35}})$ with a=0,2,4,…,20	[1]

*I* number of strata, *p*_0_ and *p*_1_ response rates in inactive/active strata, *n*_*i*_ sample size per stratum, *a* number of active strata

We would like to note that all scenario sets consider equal sample sizes across all strata. This is intended to simplify simulations. However, the optimization framework can easily be generalized to unequal sample sizes, too. Basket trial designs for unequal sample sizes can be found in the literature, e.g. [17].

For the scenario sets based on actual clinical trials, the observed response rates are known. They are listed in S1 File Section 1. After optimization of the utility functions on the scenarios mentioned in Table 1, we will calculate the performance measures of part II of the comparison study not only for the scenarios listed in the table but also for the true scenarios.

## 6 Utility functions

Under a given true scenario 𝐩 and a tuning parameter vector ϕ, we define the power in a truly active stratum *i* as the probability


$${pow}_{i}(\phi ,𝐩)=P(i\text{ detected}|\phi ,𝐩,i\text{ active})$$

and the type-I error rate (TOER) in a truly inactive stratum *i* as the probability


$${toer}_{i}(\phi ,𝐩)=P(i\text{ detected}|\phi ,𝐩,i\text{ inactive}).$$

In this context, all probabilities are “frequentist”, meaning that they are defined with respect to true binomial sampling distributions $r_{i}\sim Bin(n_{i},p_{i})$ for all *i* without being modeled on a prior distribution. However, the test decision whether a stratum is detected to be active is made according to the “Bayesian” borrowing posterior probability as defined in Eq 3. This approach is sometimes called “pragmatic Bayesianism”.

Let R⊆{1,…,I} be the set of active strata with respect to 𝐩, $R^{c}\subseteq \{1,\ldots ,I\}$ the set of inactive strata. Then, we define the experiment-wise power (EWP) as the probability


ewp(ϕ,𝐩)=P(∃i∈R:i detected|ϕ,𝐩).

Analogously, we define the family-wise error rate (FWER) as the probability


$$fwer(\phi ,𝐩)=P(\exists i\in R^{c}:i\text{ detected}|\phi ,𝐩).$$

Finally, we define the expected number of correct decisions (ECD) as


$$ecd(\phi ,𝐩)=\sum_{i\in R}P(i\text{ detected}|\phi ,𝐩)+\sum_{i\in R^{c}}P(i\text{ not detected}|\phi ,𝐩).$$

Based on these functions, we define the following utility functions. Across all definitions, $\xi_{1},\xi_{2}>0$ are penalty parameters set to 1 by default.

1. Discontinuous family-wise power-error function
  $$u_{\text{ewp}}(\phi ,p_{1},p_{2})=\left\{ \begin{array}{cc} ewp(\phi ,p_{1}) & \text{if }fwer(\phi ,p_{2})<\eta_{1},\text{ and}\\ -\xi_{1}\cdot fwer(\phi ,p_{2}) & \text{if }fwer(\phi ,p_{2})\geq \eta_{1}, \end{array}\right.$$
  usually with $p_{2}$ being the global null scenario and $\eta_{1}=0.05$.
2. Expected number of correct decisions
  $$u_{\text{ecd}}(\phi ,p_{1},p_{2})=\left\{ \begin{array}{cc} ecd(\phi ,p_{1}), & \text{if }fwer(\phi ,p_{2})<\eta_{1},\text{ and}\\ -\xi_{1}\cdot fwer(\phi ,p_{2}) & \text{if }fwer(\phi ,p_{2})\geq \eta_{1}, \end{array}\right.$$
  usually with $p_{2}$ being the global null scenario.
3. Two-level family-wise power-error function
  $$u_{2\text{ewp}}(\phi ,𝐩)=ewp(\phi ,𝐩)-\left(\xi_{1}fwer(\phi ,𝐩)+\xi_{2}(fwer(\phi ,𝐩)-\eta_{2})1(fwer(\phi ,𝐩)-\eta_{2})\right),$$
  where $\eta_{2}\in [0,1]$ is a threshold for imposing harder FWER penalty, set to 0.1 by default.
4. Two-level stratum-wise power-error function
  $$u_{2\text{pow}}(\phi ,𝐩)=\sum_{i\in R}{pow}_{i}(\phi ,𝐩)-\sum_{j\in R^{c}}(\xi_{1}{toer}_{j}(\phi ,𝐩)\;+\xi_{2}({toer}_{j}(\phi ,𝐩)-\eta_{2})1({toer}_{j}(\phi ,𝐩)-\eta_{2})),$$
  as suggested in [8].
5. Scenario-averaged versions of the above utility functions
  $${\bar{u}}_{l}(\phi ,p_{2})=\sum_{p\in \{𝐩,\ldots \}}w_{𝐩}u_{l}(\phi ,𝐩,p_{2})\;\text{for }l=\text{ewp},\text{ecd (i.e. from Item }1\text{ and }2),$$
  and
  $$\bar{u_{l}}(\phi )=\sum_{p\in \{𝐩,\ldots \}}w_{𝐩}u_{l}(\phi ,𝐩)\;\text{for }l=2\text{ewp},2\text{pow (i.e. from Item }3\text{ and }4),$$
  where {𝐩,…} is a set of scenarios of interest, e.g. the set of scenarios with number of strata *I* from Sect 5, and $w_{𝐩}$ are weights with $\sum_{𝐩}w_{𝐩}=1$, e.g. $w_{𝐩}=\frac{1}{\#\{𝐩,\ldots \}}$ for all **p**.
6. Scenario-averaged utility functions with penalty of maximal TOER inflation
  $${\bar{u}}_{l,\text{pen}}(\phi ,p_{2})=\left\{ \begin{array}{cc} {\bar{u}}_{l}(\phi ,p_{2}) & \text{if }\max_{p,j}{toer}_{j}(\phi ,𝐩)<\eta_{3},\\ -\xi_{3}\cdot \max_{𝐩,j}{toer}_{j}(\phi ,𝐩) & \text{if }\max_{p,j}{toer}_{j}(\phi ,𝐩)\geq \eta_{3}, \end{array}\right.$$
  for *l* =  ewp or ecd with the maximum defined across all p∈{𝐩,…} and $j\in R^{c}$, and
  $${\bar{u}}_{l,\text{pen}}(\phi )=\left\{ \begin{array}{cc} {\bar{u}}_{l}(\phi ) & \text{if }\max_{p,j}{toer}_{j}(\phi ,𝐩)<\eta_{3},\\ -\xi_{3}\cdot \max_{p,j}{toer}_{j}(\phi ,𝐩) & \text{if }\max_{p,j}{toer}_{j}(\phi ,𝐩)\geq \eta_{3}, \end{array}\right.$$
  for *l* =  2ewp or 2pow, where we choose $\eta_{3}=0.2$ and $\xi_{3}=1000$. In order to make the penalty work, $\xi_{3}\cdot \eta_{3}$ should be greater than the absolute value $\left|\min{\bar{u}}_{l}\right|$.

The term utility function is used as in the context of constrained optimization [18], i.e., as an objective function that is to be maximized (contrary to a loss function that would have to be minimized). In utilitarian economics and in medicine, utility may be defined as the satisfaction of individual desires by a good in general [19] or, more specifically, the improvement of individual health by a therapy, e.g. as in [20]. This meaning is not necessarily implied in the present comparison study, where the utility function is a global function across the whole trial. However, one could adapt the presented utility functions to incorporate patient utility. For example, the two-level stratum-wise power-error function could be adapted by defining $\xi_{1}=\frac{c}{g}$, were *c* is the cost of mis-detecting an inactive stratum and *g* denotes the gains from detecting a truly active stratum. A more sophisticated adaption with stratum-specific cost and gains is already discussed in [8].

We call the first four functions single-scenario utility functions. The scenario-averaged two-level stratum-wise power-error utility function ${\bar{u}}_{\text{2pow}}$ was suggested in [8], except for the fact that the authors use all possible partitions with respect to response rates whereas we allow arbitrary scenario sets. The scenario-averaged expected number of correct decisions function ${\bar{u}}_{\text{2pow}}$ emulates the optimization algorithm from [11] which in turn took the algorithm from [21]. There, the detection threshold λ is first optimized to keep FWER with respect to the global null scenario below a threshold and then the mean ECD across all scenarios is maximized subsequently.

The scenario-averaged utility functions with penalty of maximal TOER inflation are promising as they present a good compromise between research goals and regulatory requirements. In the context of borrowing from an external data source, it is an established fact that power gains using an external data source can only come at the price of type-I error inflation [5]. (This holds in the presence of a uniformly most powerful test as is the case for binary endpoints.) This shortcoming is usually accepted as a lesser evil in the context of master protocols. However, regulation may impose a constraint on maximal TOER inflation per basket, which can be taken into account by implementing the very harsh penalty $\xi_{3}$.

## 7 Optimization algorithms

For finding the optimal value (minimum or maximum) of a utility function u(·):ϕ↦u(ϕ), we will consider the optimization algorithms named in the following list. A brief explanation of the functionality of each optimization algorithm is presented below the list.

1. Bounded simulated annealing algorithm using the reflection approach for bounding the parameter space as suggested in [Section 6 of 22] – implemented in an R package developed for the purpose of this comparison study, using a start temperature of
  i. $T_{\text{start}}=100$,
  ii. $T_{\text{start}}=10$,
  iii. $T_{\text{start}}=1$,
  and one function evaluation per temperature step. Simulated annealing is inspired by a thermodynamic process, hence the physical term “temperature” as explained further below.
2. Unbounded simulated annealing algorithm using the “return NA” approach for bounding the parameter space – implemented in the R function stats::optim(), using the start temperature $T_{\text{start}}=10$ and one function evaluation per temperature step.
3. Differential evolution (DE) as implemented in the R package metaheuristicOpt, using a population size of 40, a scaling vector of 0.8 and a cross-over rate of 0.5.
4. Grey wolf optimizer (GWO) as implemented in the R package metaheuristicOpt, using a population size of 40.
5. Constrained optimization by linear approximations (COBYLA) algorithm as implemented in the R package nloptr, using stopping tolerance of 10^−6^ in the parameter space of ϕ and a stopping tolerance of 0 in the value space of u(·).
6. Grid search algorithm, searching the set of all combinations of
  λ∈{0.2,0.3,0.4,0.5,0.6,0.7,0.8,0.9}∪{0.99}∪{0.999},
  ε∈{0.0,0.5,1.0,1.5,2.0}∪{5.0,10.0,15.0,20.0,25.0}, and
  τ∈{0.0,0.1,0.2,0.3,0.4,0.5,0.6,0.7,0.8}∪{1.0}.

We will allow each algorithm to run for up to 1000 function evaluations. The grid of the grid search algorithm was chosen to use 1000 function evaluations as well. In order to put focus on parts of the grid that seemed most relevant in preliminary experiments while restricting to grid dimensions 10×10×10, we omitted some seemingly less relevant parts: λ<0.2 (i.e. test decision almost always positive), high resolution for ε>2 (i.e. sharing only with high similarity) and τ=0.9 (i.e. almost no sharing, similar to 0.8 and 1.0). If one of the simulated annealing algorithms, GWO, DE, or the COBYLA algorithm shows poor convergence after 1000 evaluations and runtime permits it, we will conduct one more run with 20 000 function evaluations. With this number of iterations, preliminary experiments with simulated annealing reached convergence, which were, however, based on an R package unfit for the analysis.

We will end this chapter with a brief explanation of the functionality of each optimization algorithm:

Simulated annealing, referred to as unbounded simulated annealing in this publication, is a physics-inspired metaheuristic optimization algorithm suggested by [23]. It builds upon the Metropolis algorithm that is also used in Markov chain Monte Carlo procedures. It is named simulated annealing as it mimics the procedure of annealing in metallurgy: There, a metal product is heated and then slowly cooled down in order to achieve a more homogeneous and stable structure within the product. The algorithm starts at a user-suggested or randomly selected initial parameter vector. In each step, the simulated annealing algorithm randomly suggests a new parameter vector near the old parameter vector. If the new vector has better utility, the current parameter vector is updated to be the new parameter vector. If it has worse utility, it is still replaced with a probability proportional to current “temperature”. At the beginning, the temperature is high in order to allow the algorithm to escape local optima. Following a prespecified temperature schedule, it is then slowly cooled down in order to find the global optimum. This algorithm was first shown to converge on finite parameter spaces in [24]. Due to the finite nature of computer memory, we can consider our parameter spaces as finite.

Bounded simulated annealing is a generalization of the simulated annealing algorithm to hypercubes of the form $\prod_{i=1}^{d}[l_{i},u_{i}]$ with lower and upper bounds *l*_*i*_ and *u*_*i*_ considered as a subset of $R^{d}$. It works identically to above-mentioned unbounded simulated annealing algorithm. Whenever a suggested parameter vector’s component $\phi_{i}$ surpasses one boundary *l*_*i*_ or *u*_*i*_, it is reflected along these boundaries until it lies in the respective interval $\left[l_{i},u_{i}\right]$. [22] suggested this modification and proved its convergence. This reflection procedure’s result can be calculated by a simple affine transformation combined with division with remainder.

Constrained optimization by linear approximation (COBYLA) is an optimization algorithm suggested by [25]. It employs a *d*-dimensional simplex in order supply linear approximations of the utility function without the need for calculating derivatives. New simplex vertices are suggested by using these linear approximations while dynamically adjusting search radius and punishment for constraint violations. The algorithm’s procedure is too complex to be described in detail but can be found in above-mentioned reference.

Differential evolution (DE) is a metaheuristic optimization algorithm that mimics genetic evolution [see 26, for an overview]. From a fixed number of candidate vectors (“the population”), donor vectors are generated by randomly adding the scaled differences of two vectors to a third vector (“mutation”). Then, new candidates (“offspring”) are generated by randomly replacing some vector components of the original candidates with components of the donor vectors (“crossover”). Finally, the new candidates replace the old candidates in the next generation if they perform better or equal (“natural selection”). Many improvements of this idea have been suggested as discussed by Das *et al*.

The grey wolf optimizer (GWO) is a metaheuristic optimization algorithm inspired by the hunting behavior and social hierarchy of grey wolves suggested by [27]. The parameter space is searched by a number of candidate vectors (“pack of wolves”) which are following the direction of the three best candidates (“alpha, beta and delta wolves”). Candidate vectors are allowed more random behavior in the beginning (“searching for prey”) and are more strictly following the best solutions in the end (“encircling the prey”). The algorithm has further been improved [28], but this improved version is not implemented in R.

Grid search is the conceptually simplest of the mentioned algorithms. For each component of the parameter vector, the user specifies a set of values of interest. Then, the algorithm simply searches all possible combinations of values for the optimal value. Grid search can be parallelized and is completely deterministic, but on the other hand, its cost grows exponentially with the number of parameters. Its precision will never be finer than the size of the mesh.

This choice of optimization algorithms is obviously not exhaustive of the abundance of available optimization algorithms. We chose these algorithms as they represent different approaches to optimization: stochastic metaheuristics with inspirations from physics, genetics and swarm behavior (simulated annealing, DE, and GWO, respectively), non-linear programming (COBYLA), and naive deterministic approaches (grid search). Both COBYLA and grid search were already applied to the optimization of clinical trial designs, see [29, 8], and [11]. Stochastic metaheuristics appear to be a good alternative as they have little requirements to the “niceness” of the targeted utility function and as they are often able to escape local minima. Availability of R implementations was also a relevant criterion in the selection of optimization algorithms.

## 8 Comparison protocol

The comparison study is divided into three parts with I. the goal of comparing optimization algorithms, II. the goal of comparing utility functions, and III. the goal of comparing the optimized parameter values to the parameter values suggested in [4] as described above. For each part of the study, we will apply the ADEMP scheme for describing simulation studies that was introduced in [9]. The ADEMP scheme was developed for describing simulation studies of statistical methods. In Part I, the methods of interest are optimization algorithms rather than statistical methods. However, the ADEMP scheme could still be adapted to match the best practices in benchmarking algorithms described in [30], namely “clarifying the reason for benchmarking” (*aim* in the ADEMP scheme), selecting an appropriate test set (*data-generating mechanism* in ADEMP) and reporting comparative measures of efficiency, reliability and quality of solution (*performance measures* in ADEMP). Some more sophisticated methods from [30] such as the choice of an exhaustive test problem set and the reporting of performance profile plots was omitted as our algorithm comparison is a quite small case study rather than a complete benchmarking of possible algorithms choices.

### 8.1. Part I: Comparison of optimization algorithms

In this first part of our comparison study, we will explore what optimization algorithm is best suited for the utility-based optimization approach. To this end, we will test the optimization algorithms on a selection of utility functions and outcome scenarios. Judging from some preliminary simulation attempts, it is expected that testing the optimization algorithms on all utility functions will take too long to be numerically feasible, see Sect 10 for a detailed explanation.

1. Aim: The goal of this part of the comparison study is to select the fastest among all reliable algorithms for optimizing the parameters of Fujikawa’s basket trial.
2. Test problems: We will consider the utility functions *scenario-averaged two-level stratum-wise power-error function* ${\bar{u}}_{\text{2ewp}}$ and *scenario-averaged expected number of correct decisions function* ${\bar{u}}_{\text{ecd}}$ and will optimize the functions on one scenario set, namely the scenario set from Sect 5 with $\left(I=4,n_{i}=20,p_{0}=0.15\right)$, yielding a total of two optimization test problems (two functions with one scenario set each). The deterministic algorithms will only be run once on each test problem, whereas the stochastic algorithms will be run $n_{\text{runs}}=50$ times on each test problem. The number of algorithm runs is justified in Sect 10 below. The seed of the first run will be 1856. As a start value for simulated annealing and COBYLA, we will choose $\phi_{\text{start}}=(\lambda_{\text{start}},\varepsilon_{\text{start}},\tau_{\text{start}})=(0.2,0.5,0)$, i.e. a test decision that is mostly positive and borrowing that takes place most of the time – this will be suboptimal in most scenarios, as it very frequently commits a type-I error.
3. Estimand/target: The target of each optimization algorithm is to find the optimal parameter combination with respect to a utility function as quickly as possible.
4. Methods: We will compare the six optimization algorithms described in Sect 7. Bounded simulated annealing will be tested with three different starting temperatures, resulting in a total of eight studied algorithms.
5. Performance measures: The following comparative measures are of interest for comparing the different algorithms. Performance measures will be presented separately per test problem. If the performance measure was measured for each run of a stochastic algorithm, the measures will be summarized using mean, standard deviation, minimal and maximal values as appropriate. For selecting the best optimization algorithm, we will use the following approach: Across all test problems, we will calculate the mean performance measures. We will begin with internal reliability. Only optimization algorithms with an internal reliability of over 99% will be considered for comparing external reliability. Only optimization algorithms with a success rate of over 99% will be considered for comparing speed. Finally, the fastest of all the remaining optimization algorithms will be considered the best algorithm.
  i. Efficiency: number of fundamental evaluations of the utility function, user CPU time, system CPU time, wall clock time, memory usage.
  ii. Internal consistency: mean resulting optimal utility function value, marginal means of the optimal parameter vector components, the component’s marginal sample standard deviations, the 95%-confidence interval of the means assuming normality, minimal and maximal values of resulting function values and parameter vector components.
  iii. External reliability: The true optimal solution is unknown, but the grid search algorithm will be used as a reference benchmark, as it is a deterministic algorithm that exhausts the whole parameter space, up to the grid’s precision. The following performance measures will then be considered: success rate of delivering an optimal utility value greater than or equal to the grid search results, 95%-confidence interval of the difference to the grid search result assuming normality, minimal and maximal difference to the grid search results.
6. Reporting: We will provide tabular presentation of the performance measures. For the stochastic algorithms, Monte Carlo standard errors (MCSE) of the performance measures will be reported wherever estimating formulae of MCSE are known. In addition, we will use box plots to visualize performance measures of interest as appropriate. Furthermore, we will generate line plots showing the convergence of the simulated annealing runs, each line representing one run, with function evaluations on the x-axis and the utility function value or one parameter on the y-axis. Finally, we will generate a four-dimensional plot of the grid search run in order to visualize the shape of each utility function: the x-axis will represent the parameter ε, the y-axis the parameter τ, plot facets will represent the parameter λ and color will represent the utility function value u(λ,ε,τ).

### 8.2. Part II: Comparison of utility functions

In the second part of the comparison study, we will explore which utility function is best-suited for the optimization of Fujikawa’s basket trial.

1. Aim: The aim of this part of the comparison study is to find the utility function which achieves the best compromise between single-stratum power and EWP on the one hand and single-stratum TOER and FWER on the other hand.
2. Data: We use the fastest reliable algorithm found in Part I of the study to optimize the tuning parameters ϕ with respect to the utility functions of interest. If the algorithm is stochastic, a seed will be fixed to 899. The functions will be optimized for the seven scenario sets introduced in Sect 5. The scenario-averaged utility functions will be averaged across all scenarios in the respective scenario set. The single-scenario utility functions, i.e., $u_{\text{l}}(\phi ,𝐩)$ with *l* =  2ewp or 2pow, will be optimized for the scenarios 𝐩= “2 of 3 active”, “2 of 4 active” and “4 of 8 active”, respectively. In the scenario sets with $\left(I=3,n_{i}=24,p_{0}=0.2\right)$ [from ] and $\left(I=4,n_{i}=20,p_{0}=0.15\right)$ [from ], all utility functions can be calculated exactly up to the precision of numerical integration using the baskexact R package [31]. Hence, this part is not actually a simulation study. The number of runs to calculate the optimal results will be 1. However, for all other scenario sets, we rely on simulation for calculating the performance measures.
3. Estimands: The estimand of each utility function is the optimal parameter vector. By applying the optimization algorithm to the respective utility function, we will receive an optimal parameter vector. The optimal parameter vector should of course be optimal with respect to the respective utility function, but should also show satisfactory performance with respect to the performance measures mentioned below.
4. Methods: The methods of interest are the twelve different utility functions mentioned in Sect 6.
5. Performance measures: For $\left(I=3,n_{i}=24,p_{0}=0.2\right)$ and $\left(I=4,n_{i}=20,p_{0}=0.15\right)$, all of the performance measures mentioned below can be calculated exactly up to the precision of numerical integration using the baskexact R package, without the necessity of Monte Carlo simulation. For all other scenarios, performance measures will be calculated using Monte Carlo simulation as implemented in the basksim package. A parameter combination is always optimal *with respect* to the scenario set, e.g. with respect to $\left(I=3,n_{i}=24,p_{0}=0.2\right)$. Performance measures will even be reported if the optimal parameter combination was optimized for another scenario in the set. For example, if $u_{\text{ewp}}(\phi ,p_{1},p_{2})$ was optimized for $p_{1}$ being the 2 of 4 strata active scenario and $p_{2}$ being the global null scenario with four strata, then we will still report the performance measures for all scenarios from the set $\left(I=4,n_{i}=20,p_{0}=0.15\right)$ mentioned in Sect 5. The following performance measures will be reported:
  i. Marginal rejection rate of the local null hypothesis in each stratum, equivalent to TOER if the stratum is inactive and power if the stratum is active,
  ii. FWER (equal to one minus the expected specificity in the terminology of [12]),
  iii. EWP (equal to the expected sensitivity in the terminology of [12]),
  iv. expected number of correct decisions,
  v. utility function value of all utility functions. Depending on the results, it may be difficult to select a clear “best choice” among the utility functions. Based on the results per scenario as well as pooled results across all scenarios, we will attempt to discuss advantages and disadvantages among the utility functions in order to suggest a “best practice”.
6. Reporting: Tabular reports of all performance measures will be provided. In addition, dot plots of the performance measures will be provided for each stratum count *I* = 3, 4 and 8. On the x-axis, the scenarios will be sorted by the number of active strata followed by the scenarios with mixed true rates. On the y-axis, the respective performance measure will be plotted.

### 8.3. Part III: Comparison of optimal parameter combinations to Fujikawa’s suggested parameter combination

Part III is an addition to the methods studied in Part II. In addition to the optimal parameter vectors obtained by utility optimization in Part II, we will also calculate the same performance measures for the parameter choice suggested in [4]. There, the shape parameter ε, the similarity cutoff τ and the detection threshold λ are suggested to be either $\phi_{\text{Fuj (i)}}=(\lambda ,\varepsilon ,\tau )=(0.99,2,0)$ or $\phi_{\text{Fuj (ii)}}=(0.99,2,0.5)$.

## 9 Further analyses

In an exploratory fashion, we will consider two further aspects in our comparison study. Firstly, Fujikawa’s basket trial design could be altered by replacing the Jensen-Shannon divergence JSD by the Hellinger distance [see e.g. 32],


$$HLD(P,Q)=1-\int_{X}\sqrt{P(x)Q(x)}\mu (dx),$$

which has the advantage that for two beta distributions, it can be calculated from basic functions without the need for numerical integration [33]:


$$HLD\left(Beta(a_{1},b_{1}),Beta(a_{2},b_{2})\right)=1-\frac{B\left(\frac{a_{1}+a_{2}}{2},\frac{b_{1}+b_{2}}{2}\right)}{\sqrt{B(a_{1},b_{1})B(a_{2},b_{2})}}.$$

We will explore whether this replacement results in a speedup of the design while at the same time maintaining a similar behavior compared to Fujikawa’s design.

Secondly, we will graphically investigate the effect of borrowing on the maximal TOER in *I* = 2 strata. One inactive basket will be kept at a stable response rate of *p*_1_ = 0.2, while we increase the response rate of the other basket from *p*_2_ = 0.2 to *p*_2_ = 1. We will then plot the response rate of *p*_2_ on the x-axis and the TOER of basket 1 on the y-axis for different combinations of ε and τ. The resulting curves may give an overview of how borrowing affects the TOER in inactive strata.

## 10 Justification of simulation size and runtime

For assessing the precision of the stochastic algorithms in Part I of our comparison study, we decided to run the algorithms for a total of $n_{\text{runs}}=50$ times. In the following Sect 10.1, we will justify the choice of this simulation size by estimating the study’s run time. For the calculation of design operating characteristics, we will use the *baskexact* package, which calculates exactly up to numerical imprecision whenever it is feasible. However, we use the Monte Carlo-based R package *basksim* [34] for estimating design characteristics in the case of large sample sizes and large stratum counts, as the *baskexact* package would take too long (see Sect 10.1 for a more detailed explanation). We use $n_{\text{MC}}=1000$ for the number of Monte Carlo-generated data sets in *basksim*. The choice of this number is justified in Sect 10.3.

For scenario sets $\left(I=3,n_{i}=24,p_{0}=0.2\right)$ and $\left(I=4,n_{i}=20,p_{0}=0.15\right)$, Part II and Part III of our comparison study are deterministic and precise up to the precision of numerical integration. Therefore, we do not conduct several runs in these parts. In other words, the simulation size for parts II and III equals 1. For the other scenario sets, the calculation of performance measures is based on Monte Carlo-generated data sets with $n_{\text{MC}}=1000$.

### 10.1 Estimation of runtime

In order to estimate the duration of our simulation, we ran a small pilot simulation: We executed the bounded simulated annealing algorithm and the grid search with 1000 iterations each for optimizing the utility function $u_{\text{ewp}}(\phi ,p_{1},p_{2})$ with respect to stratum counts *I*, per-stratum sample sizes *n*_*i*_ and the respective global null hypothesis $p_{2}$ using the R packages *baskexact* and *basksim*. The resulting run times are specified in Table 2.

**Table 2:** Run time of 1000 iterations in a pilot study.

Algorithm	*I*	*n*_*i*_	Scenario $p_{1}$	Used package	Run time
Simulated annealing	3	24	(0.2,0.2,0.5)	*baskexact*	15.64min
	4	20	(0.15,0.15,0.4,0.4)	*baskexact*	80.24min
	8	15	(0.15,0.15,0.15,0.15,0.45,0.45,0.45,0.45)	*basksim*	60.12min
Grid search	3	24	(0.2,0.2,0.5)	*baskexact*	1.03min
	4	20	(0.15,0.15,0.4,0.4)	*baskexact*	4.52min
	8	15	(0.15, 0.15, 0.15, 0.15, 0.45, 0.45, 0.45, 0.45)	*basksim*	201.67min

The *baskexact* package took 80.24min to run for $\left(I=4,n_{i}=20,p_{0}=0.15\right)$. This package calculates operating measures combinatorically and the runtime is hence influenced by the number of combinations, which is proportional to (ni)I. Therefore, it is reasonable to assume that larger scenarios, e.g. with $\left(I=8,n_{i}=15,p_{0}=0.15\right)$, would have a longer duration by several orders of magnitude more than 80.24min (e.g. $\frac{15^{8}}{20^{4}}=c$ · 10^5^), rendering any simulation infeasible. This is the reason why we will calculate the utility function for the other scenario sets with the R package *basksim*, which applies Monte Carlo simulation and can hence reduce run time. The execution of *basksim* used $n_{\text{sim}}=1000$ Monte Carlo simulations in each call to the package.

The grid search algorithm was faster than the simulated annealing algorithm by more than a factor 15 even though both algorithms ran for 1000 iterations. This is due to the fact that we used parallelization on 20 workers on our institute’s RStudio server for calculating the performance measures on the grid. Due to the sequential stochastic nature of metaheuristic optimization algorithms, parallelization of a single simulated annealing, GWO or DE run is not possible. However, we will be able to parallelize part of the $n_{\text{runs}}=50$ simulated annealing runs; hence we will also estimate a speedup by a factor 15 for Part I. In Table 3, we show a detailed explanation of the run time of Part I. The estimated run time of 15.7 days is long. However, it is still feasible while underlining the necessity to keep the example set of utility functions as small as it is.

**Table 3:** Estimation of the total run time for Part I of the comparison study.

Algorithms	Bounded simulated annealing with 3 starting temperatures, unbounded simulated annealing, DE, GWO (each of these six with $n_{\text{runs}}=50$ runs in order to investigate stochastic behavior), COBYLA, grid search
Utility functions	Scenario-averaged utility functions ${\bar{u}}_{\text{2ewp}}$ and ${\bar{u}}_{\text{ecd}}$
Scenarios	Seven scenarios with $\left(I=4,n_{i}=20,p_{0}=0.15\right)$
Run time	80.24min on a scenario with $\left(I=4,n_{i}=20,p_{0}=0.15\right)$ using the *baskexact* package
Computation clusters	20 computation kernels on the institute’s RStudio server resulting in a speedup by a factor of 15
Estimated total run time	$(6\cdot n_{\text{runs}}+2)\cdot 2\cdot 7\cdot 80.24\;\min\cdot \frac{1}{15}=15.7\;d$

The run time of Part II of our study is shown in the following Table 4. The estimation of the benefit of parallelization appears more complicated in this case: Should we parallelize across the set of 12 utility functions or across the set of up to 11 data scenarios? However, even without any parallelization, the estimated run time is 16.9 days, which would be feasible.

**Table 4:** Estimation of the total run time for Part I of the comparison study.

Algorithms	Fastest reliable algorithm selected in Part I
Utility functions	Four single-scenario utility functions, four scenario-averaged utility functions, four scenario-averaged utility functions with maximal TOER penalty
Scenarios	One scenario per scenario set for the single-scenario utility functions; 4, 7, 9, 10, 11, 5, 4 scenarios per scenario set for the scenario-averaged utility functions
Run time	15.64min on a scenario with $\left(I=3,n_{i}=24,p_{0}=0.2\right)$, 80.24min on a scenario with $\left(I=4,n_{i}=20,p_{0}=0.15\right)$ using the *baskexact* package, 60.12min on a scenario with $\left(I=8,n_{i}=15,p_{i}=0.15\right)$ with the *basksim* package, similar for large scenario sets
Computation clusters	1 computation kernel on the institute’s RStudio server resulting in no speedup
Estimated total run time	1·(4·(15.64min+80.24min+60.12min)+(4+4)·(4·15.64min+7·80.24min+(9+10+11+5+4)·60.12min))·1=16.9d

Finally note that the grid search algorithm on *I* = 8 strata is slower by a factor of 3 compared to simulated annealing. Grid search using the *basksim* package cannot use parallelization, as the *basksim* package already uses parallelization internally. However, it should optimally have about the duration as the simulated annealing algorithm, as both take the same number of iterations if executed sequentially. The unwanted slowdown may be due to a suboptimal R implementation and will be further investigated before execution of the comparison study.

### 10.2 Monte Carlo standard error of the algorithms’ precision in Part I

In Part I of the comparison study, we are interested in the precision of partly stochastic optimization algorithms. We use $n_{\text{runs}}=50$ to estimate this precision. How precise will our estimates be? The marginal sample standard deviation of the components of the resulting optimal parameter vectors is one of the most relevant performance measures of Part I of the comparison study. Assuming a normal distribution of the marginal optimal algorithm result components around the true optimal vector components, we want to keep the standard deviation of the marginal standard deviation of the vector components below a reasonable level. An unbiased estimator of the standard error of the sample standard deviation is given by


$$\hat{SD}(s)=s\cdot \frac{\Gamma (\frac{n-1}{2})}{\Gamma (n/2)}\cdot \sqrt{\frac{n-1}{2}-{\left(\frac{\Gamma (n/2)}{\Gamma (\frac{n-1}{2})}\right)}^{2}},$$

where $s=\sqrt{\frac{1}{n-1}\sum_{i=1}^{n}(X_{i}-\overset{―}{X})}$ is the sample standard deviation and $\Gamma (z)=\int_{0}^{\infty }t^{z-1}e^{-t}dt$ is the gamma function for z∈ℂ with positive real part ℜ(z)>0, see S1 File Section 2, for details. For a total of 50 algorithm runs, this means that we will achieve a standard error of $\hat{SD}(s{)}_{50}=0.10127\;\cdot \;s$, i.e., the sample standard deviation will be precise up to a standard error of little more than 10%. Regarding the already long run time, this appears acceptable.

### 10.3 Monte Carlo standard error of the design characteristics in parts I and II

In parts I and II, we will estimate the design characteristics of basket trial designs with *I* = 8 using the Monte Carlo-based package *basksim*. We want to estimate the stratum-wise power and TOER by applying the design to $n_{\text{MC}}=1000$ simulated data sets. According to [9, Table 6], the Monte Carlo standard error of a rejection rate estimate $\hat{rate}=\frac{1}{n_{\text{MC}}}\sum_{l=1}^{n_{\text{MC}}}1(p_{l}\leq \alpha )$ such as power and TOER is given by


$$\hat{SD}(\hat{rate})=\sqrt{\frac{\hat{rate}\cdot (1-\hat{rate})}{n_{\text{MC}}}}.$$

The value is maximal for $\hat{rate}=0.5$, resulting in a Monte Carlo standard error of $\hat{SD}(\hat{rate})\leq 0.016$ for $n_{\text{MC}}=1000$, i.e., a standard error of less than 2%. This seems acceptable regarding the long run time in Part I of our comparison study.

## 11 Discussion

Utility functions are a feasible and objective way of combining operating characteristics in clinical trials, which has proved useful in different contexts. So far in the context of basket trials, optimization is usually restricted to heuristic manual tuning of parameters as in [4] or to optimizing one characteristic of interest (e.g. expected number of correct decisions) while keeping type-I error rate in one scenario controlled as in [21] and [11]. The challenge of optimizing across multiple scenarios and the choice of optimization algorithm is usually not discussed. [8] suggested two types of utility functions but their choice was also not compared to other functions. Hence, our comparison study will fill a research gap in investigating both the choice of utility functions and of optimization algorithms.

Even though the framework is intended for the optimization of both Bayesian and frequentist basket trial designs, we chose frequentist performance measures as they are most common in clinical trials and hence easy to communicate despite all well-known limitations. A useful extension of the framework could be to exchange TOER and power for probability of success while replacing the different scenarios for the true response rates with an appropriate joint prior distribution.

If it proves effective and feasible, the studied optimization framework may lay the foundation for further research on optimizing basket trials, be it extensions to unbalanced sample size in the strata, multi-stage basket trials, and different Bayesian or frequentist basket trial designs.

## Supporting information

S1 File Appendix. Further information on the choice of outcome scenarios sets and on the standard error of the sample standard deviation, 7 pages
(PDF)
