# Supplementary material for: Utility-based optimization of Fujikawa’s basket trial design – Pre-specified protocol of a comparison study
Source: PLoS One. 2025 May 28;20(5):e0323097. doi: 10.1371/journal.pone.0323097 (PMC12119007; doi:10.1371/journal.pone.0323097)
Supplement: S1 File — Further information on the choice of outcome scenarios sets and on the standard error of the sample standard deviation, 7 pages (PDF) [file pone.0323097.s001.pdf]

S1 File. Appendix.

Supporting information for "Utility-based optimization of Fujikawa's basket trial design – Pre-specified protocol of a comparison study"

Lukas D Sauer[1], Alexander Ritz[2], and Meinhard Kieser[1]

[1]*Institute of Medical Biometry, Heidelberg University, Germany*
[2]*Institute of Mathematics, Clausthal University of Technology, Germany*

April 1, 2025

1 Further information on outcome scenarios

In order to study the performance of the tuning parameter combinations in the present comparison study, we want to use a wide variety of scenarios. Firstly, we are using scenarios from related methodological publications, namely Fujikawa et al. 2020 whose design we are studying, Baumann et al. 2023 whose underlying R package we are using for calculation of performance measures, and Krajewska and Rauch 2021 in anticipation of a future comparison of frequentist and Bayesian sharing techniques. The number of strata in these methodological publications vary between $I = 3$, 4 and 8 and the per-stratum sample sizes range from $n_i = 15$ to $n_i = 24$. The scenarios with low stratum and patient counts ($I = 3, n_i = 24, p_0 = 0.2$) and ($I = 4, n_i = 0, p_0 = 0.15$) enable exact calculation of performance measures (up to numerical precision of integration methods) without the necessity for Monte Carlo methods.

In order to supplement these methodologically motivated scenarios by clinically realistic scenarios, we considered the publications and published supplementary material of four different systematic literature reviews of planned and ongoing basket trials. Park et al. 2019 and Meyer et al. 2020 investigated planned and ongoing master protocols including basket trials in July 2019 and January 2020, respectively. While their search scopes and the identified trials are partially overlapping, we considered both reviews: Park et al. found more basket trials than Meyer et al., but on the other hand, Meyer et al. presented invidivual trial characteristics in the main text which made information easy and safe to extract. Kasim et al. 2023 mirrored Park et al.'s search strategy in February 2023, focussing specifically on basket trials in oncology.

Concerning search date, it is the most up-to-date of the considered reviews. The umbrella review by Haslam et al. 2023 again searched basket trials in oncology, albeit about a year earlier in March 2022. We still considered their review as they report summary data of response rates in the strata.

Table 1: Characteristics of basket trials as found in the literature. Per-group no. of patients was calculated as the total number of patients divided by the number of subgroups. Data were taken from the publications of Meyer et al. 2020, Table 1, and Haslam et al. 2023, Table 1, and from the supplementary online material of Park et al. 2019, Additional file 1, Tables S9 and S10, and of Kasim et al. 2023, Data Sheet 2.

	**Systematic review**			
**Characteristic**	**Park 2019**	**Meyer 2020**[a]	**Haslam 2023**[b]	**Kasim 2023**[c]
No. of trials, N	49	16	25	146
Total no. of patients				
Median (25–75%)	205 (90–500)	101 (95–481)	48 (30–122)	70 (35–145)
Range	12–6,452	71–11,000		0–1,609
Unknown	1	1		0
No. of strata				
Median (25–75%)	5.0 (3.5–6.5)	3.5 (1.8–4.0)		3.0 (1.0–5.0)
Range	2.0–27.0	1.0–7.0		1.0–24.0
Unknown	14	0		115
Per-stratum no. of patients				
Median (25–75%)	43 (30–71)	93 (23–145)		38 (18–61)
Range	17–514	14–2,750		6–172
Unknown	14	1		115
Response rate [%]				
Median (25–75%)			23.1 (8–30)	
Unknown	49	16		146

[a]Meyer et al. 2020 reported the planned sample size and planned number of subgroups.

[b]Only summarized data were available for Haslam et al. 2023.

[c]Kasim et al. 2023 reported the number of subgroups only for completed basket trials.

In table 1, we summarized trial characteristics as presented in the four systematic reviews. Median per-group sample size was higher in Meyer et al. 2020 compared to Park et al. 2019 and Kasim et al. 2023. As Meyer et al. reported the planned sample size, we deem the median per-group sample size in Park et al. 2019 and Kasim et al. 2023 more realistic. Considering these summary data, it is apparent that the total sample size and per-stratum sample size are usually higher in actual clinical trials than in the scenarios taken from above-mentioned methodological publications. Hence, we included four more scenario sets that are similar to actual completed basket trials registered on ClinicalTrials.gov. These four scenario sets represent trials with medium and large total sample sizes and medium and large per-stratum sample sizes.

Considering response rates, the active response rates in the methodological publications are slightly larger than the 75%-quantile reported in the systematic review by Park et al. The four realistic scenario sets complement these response rates by smaller response rates and smaller effect sizes.

1. Three-stratum scenarios from Fujikawa et al. 2020: The number of strata is $I = 3$, strata with true response rate $p_i = 0.2$ are truly inactive, strata with $p_i > 0.2$ are truly active. We will consider sample sizes of $n_i = 24$ per stratum and the following combinations of true response rates:
   a) 0 of 3 active strata: $\mathbf{p} = (0.2, 0.2, 0.2)$,
   b) 1 of 3 active strata: $\mathbf{p} = (0.2, 0.2, 0.5)$,
   c) 2 of 3 active strata: $\mathbf{p} = (0.2, 0.5, 0.5)$, and
   d) 3 of 3 active strata: $\mathbf{p} = (0.5, 0.5, 0.5)$.
2. Four-stratum scenarios from Baumann et al. 2023: The number of strata is $I = 4$, strata with true response rate $p_i = 0.15$ are truly inactive, strata with $p_i > 0.15$ are truly active. We will consider sample sizes of $n_i = 20$ per stratum and the following combinations of true response rates:
   a) 0 of 4 active strata: $\mathbf{p} = (0.15, 0.15, 0.15, 0.15)$, called "global null",
   b) 1 of 4 active strata: $\mathbf{p} = (0.15, 0.15, 0.15, 0.4)$, called "good nugget",
   c) 2 of 4 active strata $\mathbf{p} = (0.15, 0.15, 0.4, 0.4)$, called "half",
   d) 3 of 4 active strata $\mathbf{p} = (0.15, 0.4, 0.4, 0.4)$, called "bad nugget",
   e) 4 of 4 active strata $\mathbf{p} = (0.4, 0.4, 0.4, 0.4)$, called "global alternative",
   f) "one in the middle": $\mathbf{p} = (0.4, 0.4, 0.3, 0.15)$, and
   g) "linear": $\mathbf{p} = (0.15, 0.25, 0.35, 0.45)$.
3. Eight-stratum scenarios analogous to Krajewska and Rauch 2021: The number of strata is $I = 8$, strata with true response rate $p_i = 0.15$ are truly inactive, strata with $p_i > 0.15$ are truly active. We will consider sample sizes of $n_i = 15$ per stratum. This is different from the exact sample size in Krajewska and Rauch 2021, where the sample size depends on a clustering decision made at interim. However, a sample size of $n_i = 15$ is the worst case achieved when the clustering algorithm separates all strata. We will consider the following combinations of true response rates:
   a) – i) a of 8 active strata: $\mathbf{p} = (0.15, \ldots, 0.15, \underbrace{0.45, \ldots, 0.45}_{a})$ with $0 \leq a \leq 8$.

4. Scenario with medium total sample size and small effect sizes, similar to NCT02454972: In Table S5 of the supplementary Data Sheet 1 to Kasim et al., the basket trial with abovementioned ClinicalTrials.gov ID is summarized. It had $I = 9$ strata with per-stratum sample sizes ranging from 13 to 105 (median: 23) and observed per-stratum response rates of $(0.056, 0.000, 0.113, 0.143, 0.043, 0.000, 0.286, 0.065, 0.362)$. Most strata had assumed null response rates of 0.01 (corresponding to standard of care) and targeted response rates of 0.1. In our comparison study, we will consider a simplified version of this trial: We will consider a trial with $I = 9$ strata with $n_i = 23$ patients each. Strata with $p_i = 0.01$ will be considered inactive, strata with $p_i > 0.01$ will be considered truly active. For optimization, we will then consider the following data scenarios:

   a) – j) a of 9 active strata: $\mathbf{p} = (0.01, \dots, 0.01, \underbrace{0.10, \dots, 0.10}_{a})$ with $0 \leq a \leq 9$.

   When calculating the performance measures of the optimized parameter combination in part II of the comparison study, we will also consider performance measures assuming that the observed response rates from the clinical trial are the true response rates.[1]

5. Scenario with large total sample size and large number of baskets, similar to NCT02054806: In Kasim et al.'s supplementary material, another basket trial is summarized. It has $I = 20$ strata with per-stratum sample sizes ranging from 16 to 27 (median: 24) and assumed null and target response rates of 0.10 and 0.35, respectively. We will consider a simplified version with $I = 20$ strata with $n_i = 24$ patients each in which strata with $p_i = 0.10$ and $p_i > 0.10$ will be considered inactive and active, respectively. For optimization, we will then consider the following data scenarios:

   a) – k) a of 20 active strata: $\mathbf{p} = (0.10, \dots, 0.10, \underbrace{0.35, \dots, 0.35}_{a})$ with $a = 0, 2, 4, \dots, 20$.

   We will only consider even numbers in order to halve computation time. Again, we will also investigate the observed response rates

   $$(0.160, 0.174, 0.120, 0.120, 0.167, 0.043, 0.130, 0.304, 0.080, 0.042,\\ 0.200, 0.259, 0.063, 0.115, 0.000, 0.174, 0.115, 0.333, 0.091, 0.056)$$

   reported for the actual clinical trial when calculating the performance measures.

6. Scenario with medium per-stratum sample size similar to NCT01848834: In Kasim et al.'s supplementary material, yet another clinical trial is summarized. It has $I = 4$ strata with per-stratum sample sizes ranging from 32 to 60 (median: 36) and assumed null and target response rates of 0.10 and 0.35 in half of the baskets. We will consider a simplified version with $I = 4$ strata with $n_i = 36$ patients each in which strata with $p_i = 0.10$ and $p_i > 0.10$ will be considered inactive and active, respectively. For optimization, we will then consider the following data scenarios:

[1]Technical note: When using the true response rates, one needs to decide whether strata with response rates only slightly greater than p_0 are still deemed inactive. We will consider all strata with observed response rate less than $\frac{p_0+p_1}{2}$ as inactive and will set their response rates to p_0 for technical reasons. (The R packages *baskexact* and *basksim* only recognize strata with response rate precisely p_0 as inactive.)

a) – e) a of 4 active strata: $\mathbf{p} = (0.10, \dots, 0.10, \underbrace{0.35, \dots, 0.35}_{a})$ with $0 \le a \le 4$.

As before, when calculating the performance measures we will also investigate the observed response rates $(0.156, 0.167, 0.212, 0.205)$ reported for the actual clinical trial assuming that they were identical to the true response rates .

7. Scenario with large per-stratum sample size similar to NCT01631552: Kasim et al. also included the study with above-mentioned ID, whose details can be found on the website ClinicalTrials.gov, see Gilead Sciences 2024 in the references. The response to treatment was observed separately in $I = 3$ strata in 45, 54 and 108 patients, respectively (median: 54). Assumed response rates used in the planning phase were neither available at Clinical Trials.gov nor in the material provided by Kasim et al. Hence, we assumed a null response rate of 0.15 and a target response rate of 0.30. In particular, we will consider a simplified version with $I = 3$ strata with $n_i = 54$ per stratum in which strata with $p_i = 0.15$ and $p_i > 0.15$ will be considered inactive and active, respectively. For optimization, we will then consider the following data scenarios:

   a) – d) a of 3 active strata: $\mathbf{p} = (0.15, \dots, 0.15, \underbrace{0.30, \dots, 0.30}_{a})$ with $0 \le a \le 3$.

   We will again investigate the observed response rates $(0.289, 0.315, 0.333)$ as a further data scenario when calculating the performance measures.

2 Estimation of the standard error of the sample standard deviation

In the following, we present a calculation of the standard error of the sample standard deviation, following the explanation on StackExchange, see Macro 2012.

Proposition 2.1. Consider n random variables $X_i, i = 1, \dots, n$ which are independent identically normally distributed $X_i \sim N(\mu, \sigma^2)$. Furthermore, consider the usual consistent estimator of the sample standard deviation $s = \sqrt{\frac{1}{n-1} \sum_{i=1}^{n} (X_i - \bar{X})}$ and let $\Gamma(z) = \int_0^\infty t^{z-1} e^{-t} dt$ be the gamma function for $z \in \mathbb{C}$ with positive real part $\Re(z) > 0$. Then the following holds:

1. The expectation of the sample standard deviation is given by $E(s) = \frac{\sigma}{c_n}$ with the correction factor $c_n = \frac{\Gamma(\frac{n-1}{2})}{\Gamma(\frac{n}{2})} \sqrt{\frac{n-1}{2}}$.
2. $s_{\text{un}} = c_n \cdot s$ is a consistent and unbiased estimator of σ.
3. $\widehat{SD}(s) = s \cdot \sqrt{1 - \frac{1}{(c_n)^2}}$ is a consistent estimator of $SD(s)$.
4. $\widehat{SD}_{\text{un}}(s) = c_n \cdot s \cdot \sqrt{1 - \frac{1}{(c_n)^2}} = s \cdot \frac{\Gamma(\frac{n-1}{2})}{\Gamma(\frac{n}{2})} \sqrt{\frac{n-1}{2} - \left(\frac{\Gamma(\frac{n}{2})}{\Gamma(\frac{n-1}{2})} \right)^2}$.

Proof. The proof of 1. can be found in Holtzman 1950. The proof of 2. is a straightforward corollary of 1. using the linearity of expectation for proving unbiasedness and the fact that $\lim_{n\to\infty} c_n = 1$ (see Laforgia 1984) together with Slutsky's theorem for proving consistency.

Straightforward calculation using 1. shows that

$$SD(s) = \sqrt{\text{Var}(s)} = \sigma \cdot \sqrt{1 - \frac{1}{(c_n)^2}}.$$

Using the fact that s is a consistent estimator of σ and the fact that $\lim_{n\to\infty} \sqrt{1 - \frac{1}{(c_n)^2}} = 0$, this calculation implies 3. Finally, 4. follows from 2. and 3 using linearity of expectation for proving unbiasedness and once again the fact that $\lim_{n\to\infty} \sqrt{1 - \frac{1}{(c_n)^2}} = 0$ for proving consistency. □

References

Baumann, Lukas, Lukas Sauer, and Meinhard Kieser (2023). "Basket trial designs based on power priors". arXiv:2309.06988 [stat].

Fujikawa, Kei, Satoshi Teramukai, Isao Yokota, and Takashi Daimon (2020). "A Bayesian basket trial design that borrows information across strata based on the similarity between the posterior distributions of the response probability". In: *Biometrical Journal* 62.2, pp. 330–338. DOI: 10.1002/bimj.201800404.

Gilead Sciences (2024). *Study of sacituzumab govitecan-hziy (immu-132) in adults with epithelial cancer.* URL: https://clinicaltrials.gov/study/NCT01631552.

Haslam, Alyson, Timothée Olivier, Jordan Tuia, and Vinay Prasad (2023). "Umbrella review of basket trials testing a drug in tumors with actionable genetic biomarkers". In: *BMC Cancer* 23.1, p. 46. DOI: 10.1186/s12885-022-10421-w.

Holtzman, Wayne H. (1950). "The unbiased estimate of the population variance and standard deviation". In: *The American Journal of Psychology* 63.4, pp. 615–617. DOI: 10.2307/1418879.

Kasim, Adetayo, Nathan Bean, Sarah Jo Hendriksen, Tai-Tsang Chen, Helen Zhou, and Matthew A. Psioda (2023). "Basket trials in oncology: a systematic review of practices and methods, comparative analysis of innovative methods, and an appraisal of a missed opportunity". In: *Frontiers in Oncology* 13, p. 1266286. DOI: 10.3389/fonc.2023.1266286.

Krajewska, Maja and Geraldine Rauch (2021). "A new basket trial design based on clustering of homogeneous subpopulations". In: *Journal of Biopharmaceutical Statistics* 31.4, pp. 425–447. DOI: 10.1080/10543406.2021.1897993.

Laforgia, Andrea (1984). "Further inequalities for the gamma function". In: *Mathematics of Computation* 42.166, pp. 597–600. DOI: 10.1090/S0025-5718-1984-0736455-1.

Macro (2012). *Answer to "Standard deviation of standard deviation".* URL: https://stats.stackexchange.com/a/28567 (visited on 11/13/2023).

Meyer, Elias Laurin, Peter Mesenbrink, Cornelia Dunger-Baldauf, Hans-Jürgen Fülle, Ekkehard Glimm, Yuhan Li, Martin Posch, and Franz König (2020). "The evolution of master protocol clinical trial designs: A systematic literature review". In: *Clinical Therapeutics* 42.7, pp. 1330–1360. DOI: 10.1016/j.clinthera.2020.05.010.

Park, Jay J. H., Ellie Siden, Michael J. Zoratti, Louis Dron, Ofir Harari, Joel Singer, Richard T. Lester, Kristian Thorlund, and Edward J. Mills (2019). "Systematic review of basket trials, umbrella trials, and platform trials: a landscape analysis of master protocols". In: *Trials* 20.1, p. 572. DOI: 10.1186/s13063-019-3664-1.

Correspondence Lukas D Sauer, Institute of Medical Biometry, Heidelberg University, Im Neuenheimer Feld 130.3, 69120 Heidelberg, Germany. E-mail: sauer@imbi.uni-heidelberg.de
